# Immunogenicity as a Key Challenge in Tissue Engineering: Toward Clinical Translation

**DOI:** 10.34172/apb.46533

**Published:** 2026-05-10

**Authors:** Arash Abdolmaleki, Sina Jafari Dargahlou, Hussein A. Ghanimi

**Affiliations:** ^1^Department of Biophysics, Faculty of Advanced Technologies, University of Mohaghegh Ardabili, Namin, Iran; ^2^College of Nursing, University of Al-Ameed, Karbala, Iraq; ^3^Department of Clinical Laboratories, College of Applied Medical Sciences, University of Kerbala, Karbala, Iraq

## To Editor,

 Tissue engineering is widely investigated as a strategy to address organ failure and tissue loss; however, immunogenicity remains a major barrier to clinical translation. Despite advances in scaffold design, cell sourcing, and bioactive signaling, immune responses to engineered constructs continue to limit long-term function and regulatory acceptance. Immunogenicity in tissue-engineered constructs does not arise from a single component but from the interaction between scaffold chemistry, residual biological cues, and host immune context.^1^

 Decellularized extracellular matrices, for example, reduce cellular antigenicity yet frequently retain residual DNA and altered matrix epitopes capable of activating innate and adaptive immune pathways. Although thresholds such as residual DNA content below approximately 50 ng/mg dry tissue are often cited, reporting remains inconsistent with respect to fragment size, matrix modification, and analytical methodology. Consequently, comparisons across studies remain difficult and translational relevance is weakened.^1,2^

 Synthetic polymer scaffolds present a complementary challenge. While offering tunable mechanical and degradation properties, they can still elicit macrophage-driven foreign body responses, including persistent M1 polarization and foreign-body giant cell formation, which impair vascularization and integration. These responses are often assessed only at short timepoints, limiting insight into chronic immune remodeling that is critical for long-term implantation.^1,3^

 Emerging strategies aimed at mitigating immunogenicity—including immunomodulatory biomaterials delivering anti-inflammatory cues such as IL-10 or TGF-β, and CRISPR-based genome editing to reduce cellular immunogenic epitopes—remain largely preclinical.^4,5^ Such approaches raise unresolved questions regarding durability of immune modulation, off-target genetic alterations, immune recognition of edited neoepitopes, and feasibility within GMP-compliant manufacturing pipelines. Accordingly, references to “immune-privileged” engineered tissues should be confined to experimental contexts rather than implying clinical readiness.^5^

 A central obstacle to progress is the lack of harmonized, construct-specific immunogenicity assessment. Commonly used animal models incompletely capture human immune axes, particularly macrophage polarization dynamics, chronic foreign body reactions, and T-cell–mediated alloreactivity. While humanized animal models and advanced immune co-culture systems offer partial solutions, their translational value depends on clearly defined immune mechanisms and standardized readouts.^1,6^

 To accelerate clinical translation, we argue for a minimal, scaffold-class–specific framework for immunogenicity reporting. For decellularized matrices, quantitative residual DNA and protein thresholds should be reported alongside defined analytical methods.^2^ For synthetic scaffolds, longitudinal assessment of macrophage phenotype and foreign-body giant cell formation should accompany functional outcomes. For cell-containing constructs, evaluation of T-cell activation and alloreactivity is essential. Without such specificity, immunogenicity claims remain difficult to compare and insufficient for regulatory evaluation.^2,6^ Immunogenicity therefore represents not only a biological challenge, but a measurement and reporting challenge. Establishing focused, standardized immune assessment aligned with construct type and intended clinical application will be critical for translating tissue-engineered therapies from experimental promise to durable clinical reality.

## Ethical Approval

 Not applicable.
